# Evaluation of a new radiolabeled bombesin derivative with ^99m^Tc as potential targeted tumor imaging agent

**DOI:** 10.1007/s10967-013-2464-4

**Published:** 2013-03-26

**Authors:** N. Sadeghzadeh, M. Ahmadzadeh, M. Erfani

**Affiliations:** 1 Faculty of Pharmacy, Department of Radiopharmacy, Mazandaran University of Medical Sciences, Sari, Iran; 2 Nuclear Science Research School, Nuclear Science & Technology Research Institute (NSTRI), Atomic Energy Organization of Iran, Tehran, Iran

**Keywords:** GRP, Bombesin, Tumor, ^99m^Tc, Radiopeptide

## Abstract

Gastrin-releasing peptide (GRP) receptors are over-expressed in various human tumor including breast and prostate which can be targeted with bombesin for diagnosis and targeted therapy. High abdominal accumulation and the poor in vivo stability of radiolabeled bombesin analogues may represent a limitation for diagnostic imaging and targeted therapy. In this study a new bombesin derivative was labeled with ^99m^Tc via HYNIC and tricine as a coligand and investigated further. The peptide HYNIC conjugate was synthesized on a solid phase using Fmoc strategy. Labeling with ^99m^Tc was performed at 100 °C for 10 min and radiochemical analysis involved ITLC and HPLC methods. The stability of radiopeptide was checked in the presence of human serum at 37 °C up to 24 h. Internalization was studied with the human GRP receptor cell line PC-3. The Biodistribution was studied in mice. Labeling yield of >98 % was obtained to correspond a specific activity of ~80.9 GBq/μmol. Radioconjugate internalization into PC-3 cells was high and specific (15.6 ± 1.9 % at 4 h). A high and specific uptake in GRP-receptor-positive organs such as mouse tumor and pancreas (2.11 ± 0.18 and 1.78 ± 0.09 % ID/g after 1 h respectively) was also determined.

## Introduction

In recent years, small radiolabeled receptor binding peptides exhibit a great potential for receptor-imaging and tumor targeting because of their easy preparation, easy radiolabeling, rapid clearance from blood, non-target tissues, low toxicity, low immunogenicity, high affinity and specificity for receptors [1–3]. Peptide receptors are over-expressed in various human tumor cells which can be targeted with peptide-based radiopharmaceuticals for diagnosis and targeted therapy [1–4]. Bombesin (BB), a tetradecapeptide analogue of human gastrin-releasing peptide (GRP) is one of the most promising peptides which show high affinity for GRP receptor. Over-expression of GRP receptors in several malignant tumors, particularly prostate, lung, breast and colon cancers which make these receptors promising molecular targets for radiolabeled BB derivatives [2–8].

High abdominal accumulation and the poor in vivo stability of radiolabeled BB analogues may represent a limitation for diagnostic imaging and targeted therapy [9–12]. A variety of techniques are available concerning the design and development of new BB derivatives that include introduction/substitution of specific amino acids, chelating group and more important spacer chain[8, 11, 13]. All these agents have influence on radioactivity accumulation in the abdominal region and tumor/normal organ rations [11, 13].

Among the BB derivatives labeled with various radionuclides, BB (7–14) analogues seem to be one of the most BB peptides for development and design of new BB derivatives. It has been shown that the C-terminal region (Gln^7^-Trp^8^-Ala^9^-Val^10^-Gly^11^-His^12^-leu^13^-Met^14^-NH_2_) of BB is necessary for retaining receptor binding affinity and its biological activity [6, 8, 14–18]. Labeling of BB analogues with ^99m^Tc has been performed either directly or mainly indirectly via bifunctional chelator agents such as MAG_3_ (mercaptoacetyltriglycine), N_2_S_2_. DTPA (diethlenetriaminepentaacetic acid) and HYNIC (2-hydrazinonicotinamide) with or without the presence of a linker or spacer function [5, 6, 8, 11, 15, 16, 19–21]. The HYNIC-biomolecules including antibodies and peptides require coligands such as tricine and ethylenediamine diacetic acid (EDDA) for completing the coordination sphere of the technetium (V) core with allowing easy modification of the hydrophilicity and pharmacokinetics [6, 8]. It has been demonstrated in recent studies that ^99m^Tc-radiolabeling yield of HYNIC-BB derivatives using tricine as co-ligand and tricine/EDDA the exchange labeling is high [22–25]. In recent reports, utilizing various types of spacer chains has demonstrated that uncharged hydroxyl amino acids and spacer length in addition reduced kidney uptake resulted in significantly better tumor-to-tissue ration [2, 11]. Probably proteolysis of BB derivatives in plasma occur between His^12^-Leu^13^ and the slightly modified of BB derivatives have influence on stability and receptor binding affinity [11, 13, 26].

We also recently reported the preparation and evaluation of new ^99m^Tc labeled bombesin derivatives via HYNIC as a bifunctional chelating agent and tricine as co-ligand and tricine/EDDA exchange labeling [22–24]. To extend our previous study and enhance in vivo stability together with improve tumor targeting and pharmacokinetics characteristics, we chose a BB (7–14) and (d-Tyr)_2_ as a spacer to improve excretion pattern via kidney and modified d-Phe^13^ versus Leu^13^ to decrease proteolysis in plasma and increase biological activity.

Here we present data on the synthesis of [HYNIC-d-Tyr^5^-d-Tyr^6^-d-Phe^13^] BB (5–14), describe optimum conditions for radiolabeling with ^99m^Tc using tricine as coligand and in vitro/in vivo study of the radiolabeled peptide for targeting GRP receptor-positive tumors.

## Experimental

### Materials and methods

Rink amide 4-methylbenzhydrylamine (MBHA) resin and all Fmoc-protected amino acids were obtained from NovaBiochem. The prochelator HYNIC-Boc was synthesized according to Abrams et al. [27]. Other reagents were purchased from Fluka, and used without further purification. The reactive side chains of the amino acids were masked with one of the following groups: Trp, *t*-butoxycarbonyl; His, trityl; Tyr, *t*-butyl. The cell culture medium was Dulbecco’s Modified Eagle’s Medium (DMEM) supplemented with 10 % fetal bovine serum (FBS), amino acids, vitamins and penicillin/streptomycin from Gibco. Sodium pertechnetate (Na ^99m^TcO_4_) obtained from commercial ^99^Mo/^99m^Tc generator (Radioisotope Division, AEOI). Analytical reverse phase high performance liquid chromatography (RP-HPLC) was performed on a JASCO 880-PU intelligent pump HPLC system equipped with a multiwavelength detector and a flow-through Raytest–Gabi γ-detector. CC 250/4.6 Nucleosil 120-5 C18 column from Teknokroma was used for analytical HPLC, and a VP 250/10 Nucleosil 100-5 C18 column was used for semipreparative HPLC. The gradient systems consisted of 0.1 % trifluoroacetic acid/water (Solvent A) and acetonitrile (Solvent B). For analytic HPLC, Gradient I was used: 0 min 95 % A (5 % B), 5 min 95 % A (5 % B), 30 min 0 % A (100 % B), 33 min 0 % A (100 % B), 35 min 95 % A (5 % B), flow = 1 ml/min, λ = 280 nm. For semipreparative HPLC Gradient II was used: 0 min 80 % A (20 % B), 2 min 80 % A (20 % B), 17 min 50 % A (50 % B), 19 min 0 %A (100 % B), 21 min 0 % A (100 % B), 25 min 80 % A (20 % B) flow = 2 ml/min, λ = 280 nm. Mass spectrum was recorded on a HP 1100 series LC/MSD. Quantitative gamma counting was performed on an ORTEC Model 4001 M γ-system well counter.

### Synthesis

The peptide-chelator conjugate was synthesized by standard Fmoc solid phase synthesis on Rink Amide MBHA resin with substitution, 0.69 mmol/g. Coupling of each amino acid was performed in the presence of 3 mol excess of Fmoc-amino acid, 3 mol excess of *N*-hydroxybenzotriazole, 3 mol excess of Diisopropylcarbodiimide and 5 mol excess of diisopropylethylamine (DIPEA) in dimethylformamide (DMF). Coupling success was checked by the established 2,4,6-trinitrobenzenesulfonicacid test. Cleavage of the Fmoc group was achieved by repetitive treatment with 20 % piperidine in DMF. Coupling of HYNIC to peptide was performed in the presence of 1.2 mol excess of HYNIC-BOC 2.5 mol excess of (2-(7-Aza-1H-benzotriazol-1-yl)-1,1,3,3-tetramethyluronium hexafluorophosphate), 5 mol excess of DIPEA in DMF. The peptide HYNIC conjugate was removed from the resin and amino acid side chains were deprotected by treatment with a cocktail of trifluoro acetic acid, triisopropylsilane and water (95:2.5:2.5). After removing the organic solvents in vacuo, the crude product was precipitated with cold diethyl ether. The crude peptide HYNIC conjugate was dissolved in water and purified by semi-preparative (Gradient II) RP-HPLC, next the purified product was characterized by LC/MSD and analytic HPLC.

### Radiolabeling with ^99m^Tc

Radiolabeling of peptide HYNIC conjugate was performed by adding 20 μg (13.9 nmol) of the stock solution new HYNIC-BB derivative (1 mmol/l in water) and 20 mg (112 μmol) of tricine co-ligand in 0.5 mL of water. 40 μg SnCl_2_ (20 μl of 2 mg/ml SnCl_2_, 2H_2_O in nitrogen-purged 0.1 M HCl) were added to this solution. Finally, 370–1,110 MBq of ^99m^TcO_4_^−^ in 0.5 mL saline was added to the solution and incubated for 10 min at 100 °C.After cooling down to room temperature, the reaction mixture was analyzed.

### Radiochemical analysis

After cooling up to room temperature, the radiolabeling yield of the labeled peptide was determined by analytical RP-HPLC (Gradient I) and ITLC on silica gel 60 (Merck) using different mobile phases: 2-butanone for free ^99m^TcO_4_ (Rf = 1), 0.1 M sodium citrate (pH 5) to determine the non-peptide bound ^99m^Tc coligand with ^99m^TcO_4_ (Rf = 1) and methanol/1 M ammonium acetate 1/1 for ^99m^Tc colloid (Rf = 0). The radioactivity was quantified by cutting the strip (1.5 × 10 cm^2^) into 1 cm pieces and counting in a well type gamma counter.

### Human serum stability

To 1 mL of freshly prepared human serum, we added 100 μl (14.8–29.6 MBq) radiolabeled BB derivative and mixture was incubated at 37 °C. 100 μl aliquots was removed and treated with 200 μl of ethanol [11] at different times up to 24 h. Sample was centrifuged at 3,000 rpm for 15 min to precipitate serum proteins. The supernatant was filtered through a 0.20 μm pore filter and analyzed with RP-HPLC Gradient I to determine radiochemical stability.

### Cell culture

The human androgen-independent prostate carcinoma cell line PC-3 was obtained from National Cell Bank of Iran (NCBI) affiliated to Pasteur Institute of Iran. The cells were grown in DMEM supplemented with 10 % (v/v) fetal bovine serum (FBS), 1 % l-glutamine (2 mM),1 % penicillin(100 IU/ml)/streptomycin(100 μg/ml) and 1 % amphotericin B(0.25 μg/ml). The cells were incubated at 37 °C in a humidified atmosphere containing 5 % CO_2_ and were subcultured weekly detaching with trypsin/EDTA solution (0.25 %).

### Internalization assay

Medium was removed from the 6-well plates contain PC-3 cells with density of 1 million cells per well and cells were washed once with 2 ml of internalization medium (DMEM with 1 % FBS). Furthermore, 1.5 ml internalization medium was added to each well, and the plates were incubated at 37 °C for about 1 h. Afterwards, about 150 kBq (2.5 pmol total peptide mass per well) was added to the medium, and the cells were incubated at 37 °C for various time periods. To determine nonspecific internalization, we incubated cells with the radioligand in the presence of 150 μl, 1 μmol/l Bombesin. The cellular uptake was stopped at appropriate time periods (30 min, 1 h, 2 h and 4 h) by removing medium from the cells and washing twice with 1 ml of ice-cold phosphate-buffered saline (PBS). An acid wash for 10 min with a glycine buffer (pH 2.8) on ice was also performed twice. This step was to distinguish between membrane-bound (acid releasable) and internalized (acid resistant) radioligand. Finally, the cells were treated with 1 N NaOH. The culture medium, the receptor-bound and internalized fractions were measured radiometrically in a gamma counter.

### Biodistribution

Animal experiments were performed in compliance with the regulations of our institution and with generally accepted guidelines governing such a work. A suspension of human PC-3 cells (1 × 10^7^) in PBS buffer was subcutaneously injected in the right flank of each nude mouse. Seven to 10 days after inoculation, the tumors were inducted and then an activity of 20 MBq (0.35 nmol) of ^99m^Tc-Bombesin was injected via the femoral vein. In order to determine the non-specific uptake of the radiopeptides, in receptor-positive organs, a group of 3 animals were injected with 100 μg cold peptide in 50 μl saline as a co-injection with the radiopeptides (blocked animals). After 1, 4 and 24 h, the mice in groups of 3 animals were scarified, organs of interest were collected, weighed and radioactivity was measured in a gamma counter. The percentage of the injected dose per gram (% ID/g) was calculated for each tissue.

### Statistical analyses

The calculations of means and standard deviations for internalization and biodistribution were performed on Microsoft Excel. Student’s *t* test was used to determine statistical significance. Differences at the 95 % confidence level (*P* < 0.05) were considered significant.

## Results

### Synthesis

**Fig. 1:**
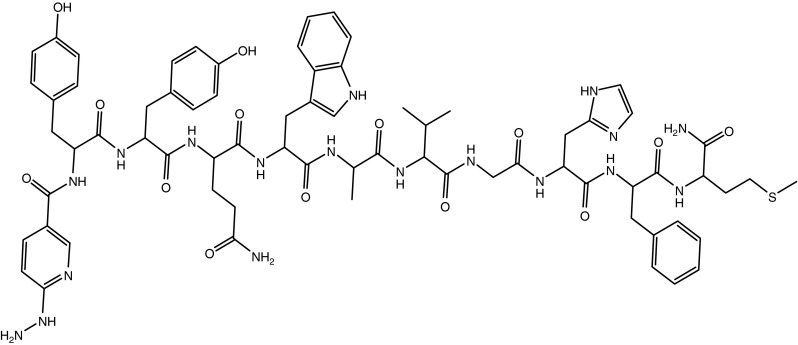
Structure of [HYNIC-d-Tyr^5^-d-Tyr^6^-d-Phe^13^] BB (5–14)

[HYNIC-d-Tyr^5^-d-Tyr^6^-d-Phe^13^] BB (5–14) (Fig. 1) was synthesized by Fmoc strategy supplying an overall yield of nearly 47 %. The composition and structural identity of HYNIC peptide was verified by LC-MSD (Table 1). The chemical purity was >99 % as confirmed by RP-HPLC method.

**Table 1:** Data for [HYNIC^0^, d-Tyr^6^, d-Trp^8^]-BZ [6–14] NH_2_ from LC/MSD analysis

Compound	Calculated mass (g)	Observed mass (g)
[HYNIC]-peptide	1434.61	1435.09 [M+H]^+^;100 %

### Radiolabling

**Fig. 2:**
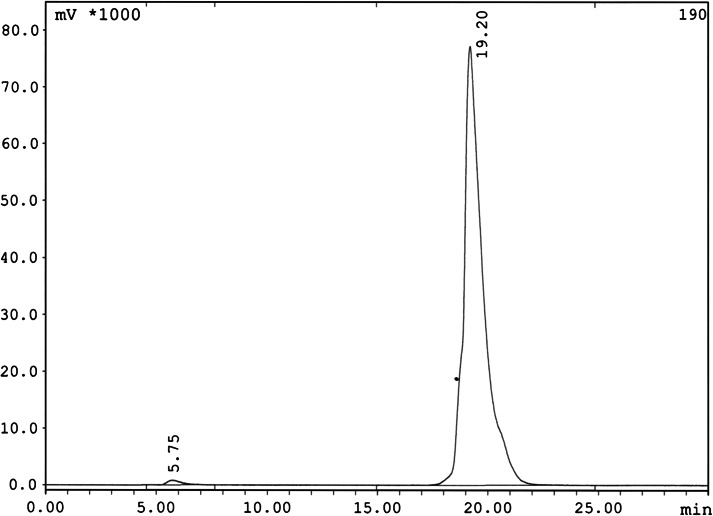
RP-HPLC profile of [^99m^Tc/tricine/HYNIC^0^, d-Tyr^5^- d-Tyr^6^-d-Phe^13^] Bombesin (5–14)

The radiochemical yield of [^99m^Tc/tricine/HYNIC^0^, d-Tyr^5^-d-Tyr^6^-d-Phe^13^] Bombesin (5–14) NH_2_, was higher than 98 % by HPLC and also ITLC at a specific activity of 81 GBq/μmol. The HPLC elution time (gradient I) were 19.20 min for ^99m^Tc-peptide and 5.72 min for ^99m^TcO_4_ (Fig. 2).

### Internalization assay and stability

**Fig. 3:**
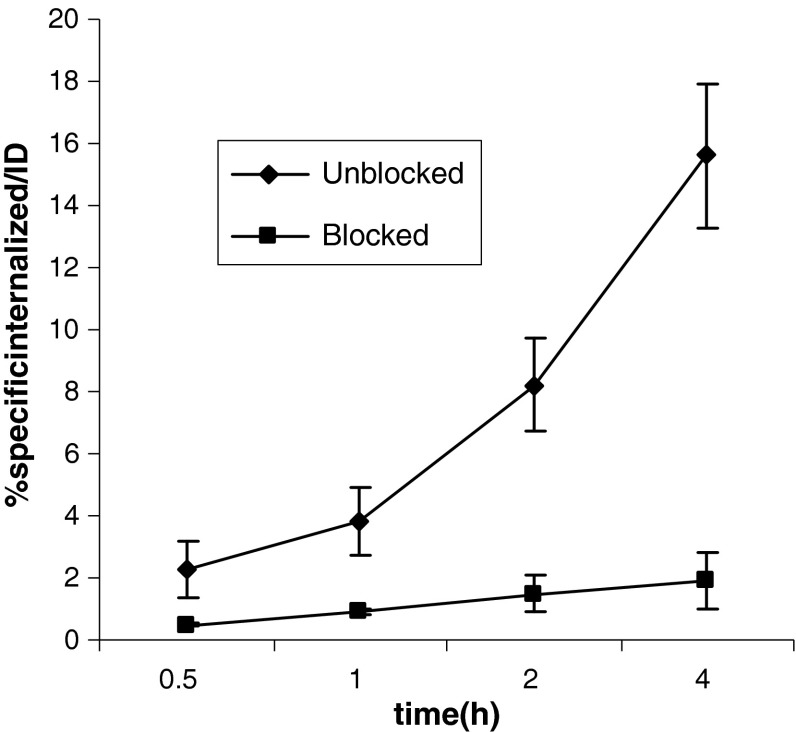
Time course internalization of [^99m^Tc/tricine/HYNIC^0^, d-Tyr^5^-d-Tyr^6^-d-Phe^13^] bombesin (5–14) in *unblocked* and *blocked* PC-3 cells. Result of three independent experiments with triplicates in each experiment

The result of the in vitro assay of the radioligand into PC-3 cell showed fast receptor-specific internalization (5.3 ± 1.1 % at 1 h and 15.6 ± 2.3 % at 4 h). As it shows the significant differences of uptake between blocked and unblocked cells in various time periods are very noticeable (*P* < 0.05) (Fig. 3). After 24 h in human serum, the radiochemical purity remained >90 %.

### Animal biodistribution studies

**Fig. 4:**
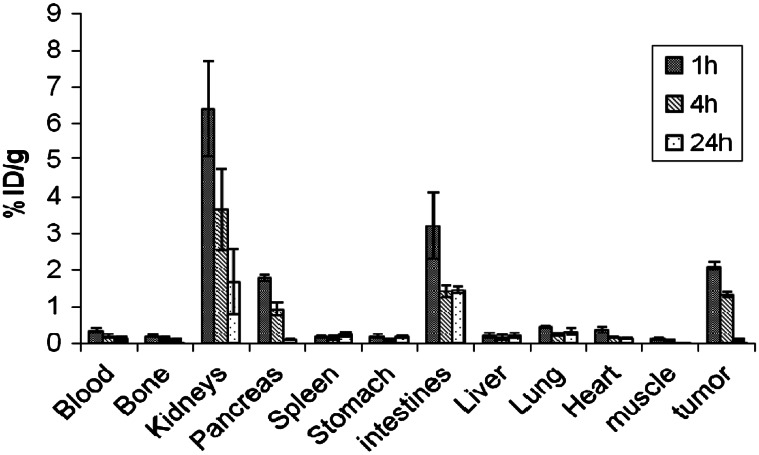
Biodistribution findings in mice (% injected dose per gram organ ± SD, n = 3)

Figure 4 shows the results of biodistribution studies. Radiopeptide exhibited a rapid clearance from the blood with 0.19 ± 0.06 % after 4 h. There was also fast clearance from the GRP receptor-negative tissues with predominantly renal excretion. New ^99m^Tc labeled BB derivative showed a high uptake of radioactivity in the PC-3 tumor and in the GRP receptor-positive organs such as the pancreas. By blocking the receptor through prior injection of cold peptide, the uptake in tumor and pancreas is diminished and this confirms the specificity of radioconjugate. Reduction uptake percentages were 82 % (1.32 vs. 0.24 % ID/g, *P* < 0.05) and 76 % (0.93 vs. 0.22 % ID/g, *P* < 0.05) respectively (Table 2). On the other hand, the uptake reduction in non-targeted tissues due to the blocking dose was not significantly.

**Table 2:** Biodistribution in mice (% injected dose per gram organ ± SD, n = 3) 4 h after administration [^99m^Tc/tricine/HYNIC^0^, d-Tyr^5^-d-Tyr^6^-d-Phe^13^] Bombesin (5–14)

Organ	Unblocked	Block
Blood	0.19 ± 0.06	0.21 ± 0.08
Bone	0.11 ± 0.05	0.14 ± 0.02
Kidneys	3.65 ± 1.12	3.85 ± 1.7
Pancreas	0.93 ± 0.17	0. 22 ± 0.12
Spleen	0.14 ± 0.06	0.17 ± 0.09
Stomach	0. 13 ± 0.03	0.11 ± 0.03
Intestines	0.96 ± 0.44	1.2 ± 0.21
Liver	0.15 ± 0.08	0.17 ± 0.04
Lung	0.23 ± 0.04	0.28 ± 0.07
Heart	0.15 ± 0.02	0.16 ± 0.02
Muscle	0.06 ± 0.02	0.07 ± 0.03
PC-3 tumor	1.32 ± 0.08	0.24 ± 0.05

## Discussion

Peptide sequences influence on tumor uptake, in vivo stability, pharmacokinetic characteristics, binding affinity for the receptors and the coordination of ^99m^Tc by HYNIC peptide conjugate [11, 13, 25, 28]. If ^99m^Tc-HYNIC peptide becomes more stable, then it may result in improved tumor targeting and body retention. On the other hand, a change in configuration would be likely to reduce the performance of the radiopharmaceutical in vivo. HYNIC makes labeling with ^99m^Tc in high specific activity possible followed by using various coligands, which permit control of the hydrophilicity and pharmacokinetics of the labeled peptide [6, 22–25, 28]. High specific activity achieves with low concentration of the HYNIC peptide conjugate. One of the most widely used coligands is tricine. Tricine gives the best radiolabeling efficiency but it has been reported that tricine as a coligand, ^99m^Tc-complex was not stable, particularly in dilute solutions, due to different bonding modalities of the hydrazine moiety of the HYNIC and the tricine coligand [6, 25, 28]. As we have previously shown that bombesin derivative [HYNIC-d-Tyr^6^-d-Trp^8^] BB (6–14) is as potential targeted tumor imaging agent [22, 23]. Therefore we extended our pervious study with a new radiolabeled bombesin derivative with sequences bombesin (7–14), D-Phe^13^ versus leu^13^ modification and (d-Tyr)_2_ as spacer to improve excretion pattern via kidney, improve binding affinity and to decrease enzymatic metabolism. In this study we used HYNIC peptide with tricine as a coligand in amounts of 20 μg and 20 mg in final volume of labeled solution respectively. We obtained high radiochemical yield (>98 %) with very low amount of ^99m^Tc-pertechnetate (<0.5 %), ^99m^Tc-radiocolloid (<1 %) and ^99m^Tc-coligand (<0.3 %). In RP-HPLC analysis, we observed a single major peak without any impurities due to isomeric forms of the new ^99m^Tc-HYNIC-conjugates. In comparison to those reports regarding ^99m^Tc-tricine-HYNIC complex instability [28, 29], our new labeled peptide conjugate was stable up to a 24 h post labeling period in the room temperature. These high labeling yield and stability may be due to optimization of condition in amount of materials, Peptide sequence and also in our labeling method.

Radiotracer showed internalization profile with increased value from 0.5 h (2.3 ± 0.9 %) to 4 h (15.6 ± 1.9 %) incubation time. The efflux rate of radiopeptide from PC-3 tumor cell after 4 h showed an acceptable intercellular trapping. Pervious works of BB derivatives also demonstrate internalization and receptor mediated trapping of labeled compounds [4, 21]. Compare with our pervious compound [^99m^Tc-tricine-HYNIC^0^-d-Tyr^6^-d-Trp^8^] BB (6–14) [22, 23], this new derivative showed higher rate of internalization after 4 h in PC-3 (15.6 ± 1.9 vs. 10.7 ± 1.2 %).It could be due to d-Phe^13^ versus leu^13^ modification and replacement of (d-Tyr)_2_ as spacer instead of d-Tyr^6^. As liolios et al. [11] reported that the incorporation of positively and negatively charged the amino acid spacer decrease the binding affinity of the BB analogue for GRP receptor. In the present study, it seems that uncharged the amino acid composed from tyrosine increases binding affinity of new BB analogue.

The results of biodistribution showed fast blood clearance of radiopeptide with <0.19 % ID/g and rapid excretion performed mainly by renal pathway at 4 h. In addition, in vivo studies exhibited low abdominal accumulation and significant and specific uptake of radioconjugate in tumor and pancreas. The highest non-specific uptake was found in kidneys. A significant uptake of radioactivity was observed in the pancreas which expresses GRP receptors. The specificity of radioconjugate was confirmed by blocking the receptor through prior injection of cold peptide.

Garayoa et al. [30] reported that uncharged hydroxyl linker as serine improved the biodistribution and resulting in increase in the tumor uptake. Compare with our pervious study [22, 23] the uptake of radiopeptide in tumor and pancreas increased (2.11 vs. 1.12 % ID/g, *P* < 0.05 and 1.78 vs. 1.04 % ID/g, *P* < 0.05 at 1 h) respectively. These results indicate that the charge in linker might be a key factor for determining the pharmacological characteristics of BB derivatives. As the most of BB derivatives exhibit high abdominal accumulation which may represent a problem in their clinical for diagnostic imaging and targeted therapy [12]. Our compound with modified lipophilicity has a good improvement in renal excretion, significant and specific tumor uptake.

## Conclusions

In general, this study performed for new [HYNIC-d-Tyr^5^-d-Tyr^6^-d-Phe^13^] BB (5–14) derivative showed that hydrophilicity, charge of spacer and sequence of peptide influenced on the bidistribution and the affinity. In this study, labeling of [HYNIC-d-Tyr^5^-d-Tyr^6^-d-Phe^13^] BB (5–14) with ^99m^Tc was completed within a very short time by using tricine coligand in high specific activity (~80.9 GBq/μmol) which makes it an ideal conjugate for usage in clinical nuclear medicine laboratories. Furthermore, this labeled peptide conjugate demonstrated excellent radiochemical stability even up to 24 h post labeling. Our new radiopeptide had a specific cell binding and internalization followed by a good stability in human serum at 37 °C for at least 24 h and no significant impurities were detected by HPLC. The prepared radiopeptide showed a high accumulation of radioactivity in tumor and pancreas as a positive GRP receptors targeted tissue followed by excretion via the kidneys. These promising Characteristics make our new designed labeled peptide conjugate as a very suitable candidate for diagnostic imaging of GRP receptor-positive tumors.
